# Altered Expression of Thyroid- and Calcium Ion Channels-Related Genes in Rat Testes by Short-Term Exposure to Commercial Herbicides Paraquat or 2,4-D

**DOI:** 10.3390/jox14040081

**Published:** 2024-10-09

**Authors:** Enoch Luis, Vanessa Conde-Maldonado, Edelmira García-Nieto, Libertad Juárez-Santacruz, Mayvi Alvarado, Arely Anaya-Hernández

**Affiliations:** 1 Investigadores por México CONAHCYT—Instituto de Fisiología Celular, Universidad Nacional Autónoma de México, Circuito Exterior s/n, C.U., Ciudad de México 04510, Mexico; enoch@ifc.unam.mx; 2 Laboratorio Nacional de Canalopatías, Instituto de Fisiología Celular, Universidad Nacional Autónoma de México, Circuito Exterior s/n, C.U., Ciudad de México 04510, Mexico; 3 Maestría en Ciencias en Sistemas del Ambiente, Centro de Investigación en Genética y Ambiente, Universidad Autónoma de Tlaxcala, Tlaxcala de Xicohténcatl 90000, Mexico; codemv21@gmail.com (V.C.-M.); edelmira.garcianieto@uatx.mx (E.G.-N.); liber_05@yahoo.com.mx (L.J.-S.); 4 Laboratorio de Toxicología y Química Ambiental, Centro de Investigación en Genética y Ambiente, Universidad Autónoma de Tlaxcala, Km 10.5 Autopista Tlaxcala-San Martín, Ixtacuixtla 90120, Tlaxcala, Mexico; 5 Instituto de Neuroetología, Universidad Veracruzana, Xalapa 91190, Veracruz, Mexico; malvarado@uv.mx

**Keywords:** herbicides, endocrine disruptor, thyroid disruption, reproductive toxicity, male infertility

## Abstract

Exposure to pesticides such as paraquat and 2,4-dichlorophenoxyacetic acid (2,4-D) has been linked to harmful health effects, including alterations in male reproduction. Both herbicides are widely used in developing countries and have been associated with reproductive alterations, such as disruption of spermatogenesis and steroidogenesis. The thyroid axis and Ca^2+^-permeable ion channels play a key role in these processes, and their disruption can lead to reproductive issues and even infertility. This study evaluated the short-term effects of exposure to commercial herbicides based on paraquat and 2,4-D on gene expression in rat testes. At the molecular level, exposure to paraquat increased the expression of the thyroid hormone transporters monocarboxylate transporter 8 (*Mct8*) and organic anion-transporting polypeptide 1C1 (*Oatp1c1*) and the thyroid receptor alpha (*TRα*), suggesting a possible endocrine disruption. However, it did not alter the expression of the sperm-associated cation channels (*CatSper1-2*) or vanilloid receptor-related osmotically activated channel (*Trpv4*) related to sperm motility. In contrast, exposure to 2,4-D reduced the expression of the *Mct10* transporter, *Dio2* deiodinase, and *CatSper1*, which could affect both the availability of T3 in testicular cells and sperm quality, consistent with previous studies. However, 2,4-D did not affect the expression of *CatSper2* or *Trpv4*. Deregulation of gene expression could explain the alterations in male reproductive processes reported by exposure to paraquat and 2,4-D. These thyroid hormone-related genes can serve as molecular biomarkers to assess endocrine disruption due to exposure to these herbicides, aiding in evaluating the health risks of pesticides.

## 1. Introduction

Pesticide exposition, such as pyrethroids, organophosphates, phenoxyacetic acids, carbamates, organochlorines, and a mixture of these compounds, has been linked with harmful effects on the health of organisms, including humans [1,2]. The most used pesticides in developing countries are paraquat and 2,4-dichlorophenoxyacetic acid. Among these herbicides’ common side effects are male reproductive alterations [3,4,5].

Paraquat (1,1′-dimethyl-4,4′-bipyridinium dichloride; Table 1), classified as a quaternary ammonium bipyridylium group herbicide, has been used since the 1960s. It is characterized as a broad-spectrum herbicide with rapid action, acting on contact and being non-selective. Paraquat is a highly toxic compound for both humans and animals [6,7]. It is classified in toxicological terms as moderately dangerous (Category II toxins; oral route) and slightly toxic (Category III toxins) by the dermal route [8]. Despite being banned in over 67 countries (European Union, Kuwait, China, among others), it is still used in over 130 countries, including Thailand, Fiji, Samoa, the United States, Guatemala, Belize, Nicaragua, Paraguay, Colombia, and Mexico [8,9,10]. Paraquat use is associated with a high annual mortality rate, reaching 54% in the United States, 74% in France, and 80% in Asia, with the latter region having the highest incidence due to its use for suicidal purposes [10]. Paraquat exerts its toxic effects primarily through its redox cycle, increasing the formation of free radicals and oxidative stress in organisms and finally causing cell death [11].

In rat experiments, exposure to paraquat has been shown to reduce serum testosterone levels as well as the mRNA levels of key enzymes involved in steroidogenesis (carried out by Leydig cells), such as *Hsd17b3*, *Srd5a1*, *Hsd11b1*, *Cyp11a1*, *Cyp17a1*, and *Hsd11b1* [12]. This compound has also been shown to decrease sperm count, although it does not affect the number of Leydig cells. Additionally, a significant decrease in sperm motility and viability and increased teratospermia have been observed. Histologically, paraquat increases lipid peroxidation and apoptosis in the testes, especially for germ cells [13]. At the organism level, decreases in body weight and testicular and epididymal weights have been observed [14].

On the other hand, 2,4-dichlorophenoxyacetic acid (2,4-D; Table 1) is a member of the phenoxyacetic herbicide group. These herbicides are considered moderately toxic and classified as Group 2B (possible carcinogen) by the International Agency for Cancer Research [15]. They are used as auxins (synthetic plant hormones) in various crops to control broadleaf weeds. Their action mode is analogous to natural auxin hormones [16]. The effects of exposure to 2,4-D have been widely studied, with numerous controversies regarding their impact on human health [17].

Epidemiological studies have associated exposure to this compound with conditions such as asthenospermia, azoospermia, and teratospermia [18]. In rats, exposure to 2,4-D has decreased body weight and those of the testes, seminal vesicles, and prostate. Histologically, intracellular spaces, tissue loss, and seminiferous tubule atrophy have been observed [19]. Additionally, reductions in the count and motility of sperm and testosterone serum levels have been evidenced [20]. Evaluations of human sperm in vitro have shown that 2,4-D does not affect viability, capacitation, or acrosomal reactions but does inhibit the sperm total, progressive motility, and progesterone-induced capacitation [21].

Although the studies mentioned above highlight that both paraquat and 2,4-D exposure can disrupt testicular function and thus affect male fertility, it is necessary to investigate other molecular biomarkers which could be involved with these pathological effects. Since both herbicides, paraquat, and 2,4-D interfere with steroidogenesis and spermatogenesis (especially sperm motility), genes associated with thyroid hormone action and regulating Ca^2+^-permeable ion channels could provide information on these reproductive alterations.

It is well known that the activity of the thyroid hormones thyroxine (T4) and triiodo-tyrosine (T3) is vital for the processes of spermatogenesis and steroidogenesis, directly influencing testicular physiology and therefore male fertility [22,23,24]. The levels of thyroid hormones circulating in the blood are essential to exerting their tissue action. However, to carry out their correct function, they require their transporters, receptors, and deiodinases, among others, which determine the appropriate action of thyroid hormones for each target cell, including male reproductive cells [25,26]. Although exposure to paraquat and 2,4-D in humans and rats has been observed to alter the serum levels of thyroid hormones such as T4, T3, and thyrotropin (TSH) [27,28,29], the impact of these herbicides on thyroid hormone transporters, deiodinases, and receptors has not yet been evaluated.

In addition to hormones, ion channels are essential links between transient changes in the membrane potential and various cellular responses. Cellular signaling in testicular cells can be initiated and maintained by the activity of voltage-activated, pH-activated, non-selective cation channels, as well as several ligand-activated channels responsible for regulating multiple processes, including sperm motility, the acrosomal reaction, and other diverse physiological processes key to successful fertilization [30,31,32]. Among the ion channels that participate in hyperactivated motility and are vital for the survival and fertility of sperm are the sperm-specific Ca^2+^-permeable channel (CatSper) and the vanilloid receptor-related osmotically activated channel (transient receptor potential channel subfamily V member 4 (Trpv4)) [31,32].

Regarding the effects of paraquat and 2,4-D on calcium-permeable channels, no studies have reported their impact on the CatSper or Trpv4 channels. However, it is known that heavy metals such as lead, mercury, and cadmium [33,34], as well as other environmental contaminants like bisphenol [35,36], p,p’DDE [37], pentachlorophenol [38], and dioxins [39], can alter the functionality of the CatSper, which can result in decreased hyperactivation and possibly lead to fertility issues.

Therefore, the present study aims to evaluate the testicular gene expression of the thyroid hormone receptors (*TRα* and *TRβ)*, deiodinases (*Dio2* and *Dio3*), and thyroid hormone transporters (*Mct8*, *Mct10*, and *Oatp1c1*) as well as the gene expression of the ion channels *CatSper1*, *CatSper2*, and *Trpv4* in response to short-term exposure to paraquat or 2,4-D in male rats.

## 2. Materials and Methods

### 2.1. Animals

Adult male Wistar rats (*Rattus norvegicus*) aged 2 months and weighing 180–200 g were obtained from the Center for Research and Advanced Studies of the National Polytechnic Institute (CINVESTAV, Zacatenco Unit). The animals were housed at the Center for Research in Genetics and Environment (Autonomous University of Tlaxcala) under standard conditions (12 h light and 12 h dark cycle, with lights on at 8:00 am; 22 ± 2 °C), grouped in collective acrylic boxes, provided with Purina rat chow and water ad libitum, and subjected to a 4 week acclimatization period. All protocols and procedures involving animals were conducted following the guidelines of the Mexican Official Standard for the Production, Care, and Use of Laboratory Animals (NOM-062-ZOO-199) under the approval and supervision of Universidad Autónoma de Tlaxcala, whose internal Bioethical Committee carefully reviewed and approved this research protocol.

### 2.2. Short-Term Exposure to Commercial Pesticide Paraquat or 2,4-D

**Figure 1:**
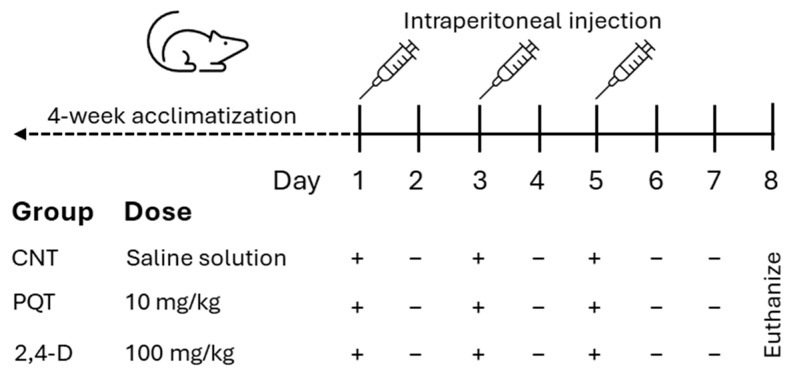
Experimental design of short-term exposure to commercial herbicides paraquat (PQT) or 2,4-D, showing herbicide administration (+) or no herbicide administration (−).

After the acclimatization period, the rats were randomly assigned to three experimental groups: the control group (CNT; *n* = 8), the paraquat group (PQT; *n* = 8), and the 2,4-dichlorophenoxyacetic acid group (2,4-D; *n* = 8). The commercial pesticides Lucaquat (25% paraquat) and Desmonte A (41% 2,4-D) were used, and they were diluted in saline solution to achieve final doses of 10 and 100 mg/kg body weight (b.w.), respectively (200 µL final volume). The CNT group received an intraperitoneal injection (i.p.) of the vehicle (200 µL saline solution). Paraquat or 2,4-D administration was performed i.p. three times per 10 and 100 mg/kg at 48 h intervals to a final dose of 30 and 300 mg/kg b.w. (Figure 1).

### 2.3. Tissue Extraction

The rats were euthanized with an overdose of sodium pentobarbital (50 mg/kg i.p., Pisa) 72 h after the last administration of pesticides or the vehicle. The testes and epididymis were removed, weighed, immediately placed in liquid nitrogen, and stored at −80 °C until further use.

### 2.4. Reverse Transcription Followed by Semiquantitative PCR (RT-PCR)

The RNA from the left testes rats (six per group) was isolated using TRI reagent (Sigma-Aldrich, St. Louis, MO, USA) and treated with DNase (RQ1 RNase-Free DNase; Promega Corporation, Madison, WI, USA) according to a previous report by Luis et al. (2019) [40]. The reverse transcription reaction was performed in a Verity Thermal Cycler (Applied Biosystems, Foster City, CA, USA) in a single run using M-MLV a reverse transcriptase (Promega, Madison, WI, USA).

Analysis of the mRNA expression of the thyroid-related genes (*TRα*, *TRβ*, *Dio2-3*, *Mct8-10*, and *Oatp1c1*) and ion channel genes (*CatSper1-2* and *Trpv4*) was performed in triplicate using primers obtained from the previous reports (Table 2) and purchased from Sigma-Aldrich (USA). PCRs were carried out with Taq DNA polymerase (Sigma-Aldrich, USA) and specific primers. Negative controls were included where cDNA was omitted. The PCR products were visualized on 2.5% agarose gels stained with ethidium bromide and analyzed using a UV-transilluminator (UVP, Upland, CA, USA). The relative expression levels of several genes were determined by densitometry using ImageJ software, version 1.54i (NIH, Bethesda, MD, USA), and the results were normalized to *Ppia* gene expression.

### 2.5. Statical Analysis

The normality of all relative gene expression data was assessed using the Shapiro–Wilk (S-W) test and subsequently analyzed using a *t*-test (comparing each exposed group versus the control group). A one-way ANOVA test followed by a post hoc Tukey test was used for multiple comparisons (comparing the relative levels of expression of genes intra-group). The results are depicted as the mean (n = 6 per group) ± standard error of the mean (SEM) from three experiments for each gene. Statistical analysis was performed using GraphPad Prism version 8.0.1 software (La Jolla, CA, USA), with significance set at *p* ≤ 0.05.

## 3. Results

### 3.1. Effect of Paraquat or 2,4-D on Body, Testes, and Epididymis Weights

The animals in the CNT group and those exposed to the herbicides PQT and 2,4-D showed similar weights at the beginning of the treatment (225.3 ± 30.3; 214.1 ± 17.6; and 219.6 ± 36.6 g, respectively; ANOVA, *p* = 0.7316). At the end of the treatment, the animals in the control group were the only ones to show a significant increase in body weight (*p* = 0.0207). In contrast, the animals in the PQT group showed a slight reduction in body weight. Meanwhile, the animals in the 2,4-D group maintained similar weights before and after the treatment. Only paraquat exposure decreased the body weights of the male rats. Neither herbicides affected the testes or epididymis weight (Table 3).

### 3.2. Effect of Paraquat or 2,4-D on the Expression of Thyroid Hormone-Related Genes

#### 3.2.1. Thyroid Hormone Transporters

**Figure 2:**
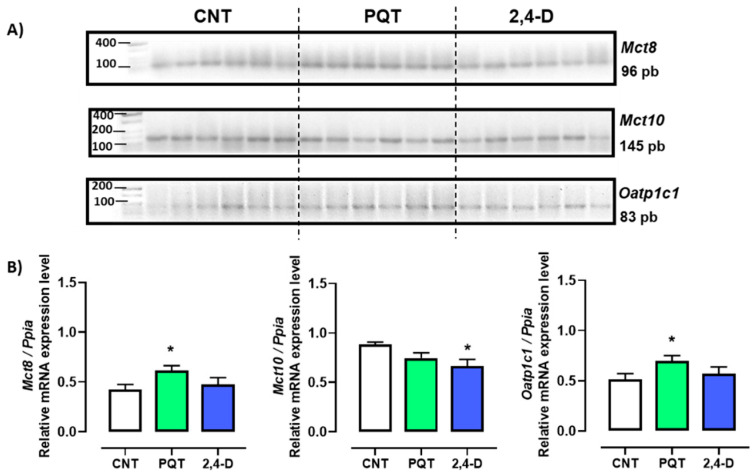
Effect of short-term exposure to paraquat or 2,4-D on the expression of thyroid hormone transporters. (**A**) Representative image of 2.5% agarose gel electrophoresis stained with ethidium bromide. Amplified bands of thyroid hormone transporters in rat testes of control (CNT, n = 6), paraquat (PQT, n = 6), and 2,4-D (n = 6) groups. (**B**) Comparison between groups of transporters’ expression relative to *Ppia* gene expression. Means ± SEM are shown. Statistical analysis was performed using the normality test (S-W) and *t*-test (* *p* ≤ 0.05).

The expression of thyroid hormone transporters *Mct8*, *Mct10*, and *Oatp1c1* was evaluated, and among these three transporters, the most abundant gene expressed in the testes of the CNT group was that of the *Mct10* transporter (0.4245 ± 0.0505; 0.8807 ± 0.0277; and 0.5135 ± 0.0581 a.u., respectively; ANOVA and Tukey’s multiple comparison test, *p* < 0.0001). Conversely, animals exposed to the herbicide PQT showed similar expression in all three transporters *Mct8*, *Mct10*, and *Oatp1c1* (0.6150 ± 0.0489; 0.7446 ± 0.0547; and 0.6956 ± 0.0547 a.u., respectively; ANOVA, *p* = 0.2432). Similar to the PQT group, the 2,4-D group showed the same expression for the three transporters (0.4740 ± 0.0691; 0.6641 ± 0.0691; and 0.5721 ± 0.0659 a.u., respectively; ANOVA, *p* = 0.1711). The transporters *Mct8* and *Oatp1c1* exhibited a similar expression pattern across all three groups (Figure 2).

In the rats exposed to PQT, there was a significant increase in gene expression of the transporters *Mct8* and *Oatp1c1* compared with the CNT group animals (*p* = 0.0207 and 0.0439, respectively). Conversely, the rats exposed to 2,4-D showed similar gene expression levels of *Mct8* and *Oatp1c1* (*p* = 0.5864 and *p* = 0.5254, respectively) and lower gene expression of *Mct10* (*p* = 0.0194) compared with the CNT group animals (Figure 2).

#### 3.2.2. Deiodinases

**Figure 3:**
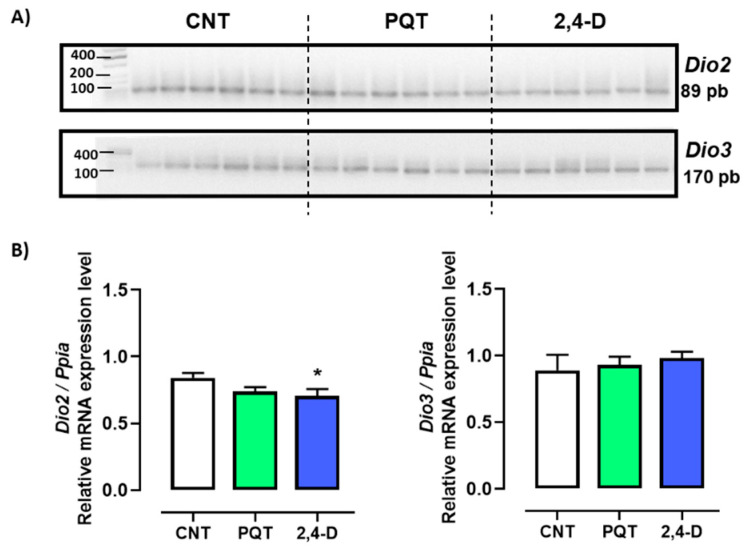
Effect of short-term exposure to paraquat or 2,4-D on the expression of deiodinases. (**A**) Representative image of 2.5% agarose gel electrophoresis stained with ethidium bromide. Amplified bands of *Dio2* and *Dio3* in rat testes of control (CNT, n = 6), paraquat (PQT, n = 6), and 2,4-D (n = 6) groups. (**B**) Comparison between groups of *Dio2* and *Dio3* expression relative to *Ppia* gene expression. Means ± SEM are shown. Statistical analysis was performed using a normality test (S-W) and *t*-test (* *p* ≤ 0.05).

The relative gene expression of both deiodinases *Dio2* and *Dio3* were similar in the testes of rats from the CNT group (0.8412 ± 0.0357 versus 0.8898 ± 0.1169 u.a., respectively; *p* = 0.6989). However, the relative expression of *Dio2* compared with *Dio3* in the rats exposed to paraquat was statistically lower (0.7389 ± 0.0327 versus 0.9307 ± 0.0613 u.a., respectively; *p* = 0.0173). Similarly, the 2,4-D group showed lower expression of *Dio2* versus *Dio3* (0.7067 ± 0.0504 versus 0.9823 ± 0.0482 u.a., respectively; *p* = 0.0023) (Figure 3).

Exposure to PQT did not affect the expression of either deiodinases compared with the CNT group (*Dio2: p* = 0.0584; *Dio3: p* = 0.7525). However, exposure to 2,4-D decreased only the relative gene expression of *Dio2* compared with the CNT group (*p* = 0.0445) (Figure 3).

#### 3.2.3. Thyroid Hormone Receptors

The expression of thyroid hormone receptors *TRα* and *TRβ* in the rat testes was evaluated. Gene expression of *TRβ* was found to be scarce or almost negligible in all animals analyzed, making its quantification unfeasible.

**Figure 4:**
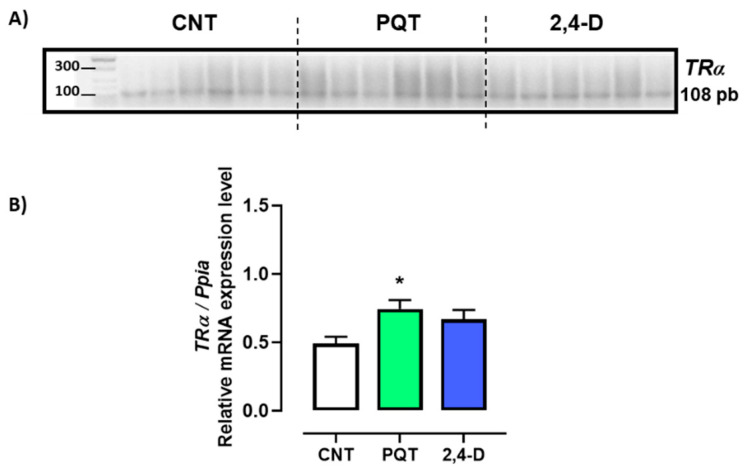
Effect of short-term exposure to paraquat or 2,4-D on the relative expression of thyroid hormone receptor alpha (*TRα*). (**A**) Representative image of 2.5% agarose gel electrophoresis stained with ethidium bromide. Amplified bands of *TRα* in rat testes of control (CNT, n = 6), paraquat (PQT, n = 6), and 2,4-D (n = 6) groups. (**B**) Comparison between groups of *TRα* expression relative to *Ppia* gene expression. Means ± SEM are shown. Statistical analysis was performed using a normality test (S-W) and *t*-test (* *p* ≤ 0.05).

The animals exposed to PQT exhibited significantly higher relative gene expression of *TRα* in the testes than those in the CNT group (0.7420 ± 0.0660 versus 0.4895 ± 0.0494 a.u., respectively; *p* = 0.0127). Exposure to 2,4-D did not significantly alter the gene expression of *TRα* (0.6673 ± 0.0676 a.u.; *p* = 0.0641) (Figure 4).

### 3.3. Effect of Paraquat or 2,4-D on the Expression of Ion Channels Involved in Sperm Flagellar Hyperactivation

In the rat testes, the gene expression of three Ca^2+^-permeable ion channels—*CatSper1*, *CatSper2*, and *Trpv4*—was observed (Figure 4-A). In the testes of the CNT group, the most abundant genes expressed were *CatSper1* and *Catsper2* (0.9597 ± 0.0700 and 1.031 ± 0.0547 a.u., respectively) versus *Trpv4* (0.7718 ± 0.0554 a.u.; ANOVA and Tukey’s multiple comparison test, *p* = 0.0231).

**Figure 5:**
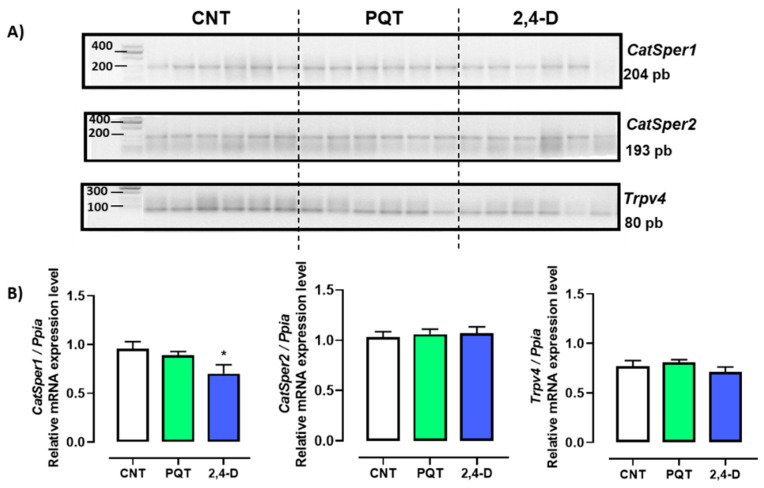
Effect of short-term exposure to paraquat or 2,4-D on the expression of ion channels. (**A**) Representative image of 2.5% agarose gel electrophoresis stained with ethidium bromide. Amplified bands of *CatSper1*, *CatSper2*, and *Trpv4* in rat testes of control (CNT, n = 6), paraquat (PQT, n = 6), and 2,4-D (n = 6) groups. (**B**) Comparison between groups of ion channel expression relative to *Ppia* gene expression. Means ± SEM are shown. Statistical analysis was performed using a normality test (S-W) and *t*-test (* *p* ≤ 0.05).

Exposure to paraquat did not affect the gene expression of any of the sperm-specific channels (*CatSper1* (0.8894 ± 0.0390 a.u., *p* = 0.3816), *CatSper2* (1.0600 ± 0.0501 a.u., *p* = 0.6986), or *Trpv4* (0.8091 ± 0.0259 a.u., *p* = 0.5349)) compared with the CNT group. On the other hand, exposure to 2,4-D reduced the gene expression of *CatSper1* (0.7027 ± 0.0893 a.u., *p* = 0.0495) but not *CatSper2* (1.0680 ± 0.0657 a.u., *p* = 0.6775) or *Trpv4* (0.7106 ± 0.0515 a.u., *p* = 0.4357) compared with the CNT group (Figure 5).

## 4. Discussion

Currently, approximately 3.7 million tons of pesticides are used worldwide, 52.5% of which are herbicides. The five countries which consume the most herbicides globally are Brazil, the United States, Argentina, China, and Canada [44]. Herbicides are applied to increase crop productivity. However, they can accumulate in different parts of plants over time. Additionally, they can be deposited in the soil directly from their application to crops or indirectly through airborne transport from other areas. Once in the soil, herbicides can adsorb due to their high affinity for soil particles, or they can be transported to surface waters through run-off or leaching and may even infiltrate into groundwater. Herbicide application can also generate vapors, contaminating the air and eventually settling in the soil or surface waters [45,46]. Environmental exposure to herbicides can occur in various ways, affecting animals and humans. In animals, the most common routes include accidental ingestion of contaminated water, inhalation of herbicide aerosols during application, or dermal contact with treated plants. In agricultural environments, it is common for animals to be exposed to herbicides by consuming contaminated vegetation or water from sources near treated areas. In humans, the main routes of exposure include ingestion of contaminated food, inhalation, dermal contact, and in some cases accidental ingestion [47,48,49]. In humans, these routes of exposure, whether direct (during manufacturing, transport, storage, or application) or indirect (through environmental contamination), are particularly concerning due to their potential health impacts.

Much of what is known about the toxicity of herbicides in mammals comes from laboratory studies on mice and rats. These animal models are widely used in toxicological studies due to their physiological similarity to other mammals, making them helpful in assessing toxicity mechanisms and systemic responses [50,51]. Previous research has used rats to examine the effects of exposure to paraquat and 2,4-D on reproductive physiology, providing us with a solid foundation for the development of this study [12,13,14,20,52].

Although the effects on reproduction due to exposure to paraquat or 2,4-D have been extensively studied in humans and laboratory mammal models, relatively little is known about their impact on other vertebrate wildlife species or livestock [53,54]. In humans, for instance, studies have reported a decrease in sperm quality, hormonal imbalances, and testicular cytotoxicity, highlighting the severe implications of herbicide exposure on reproductive health [18,48,55,56].

The subacute and chronic effects of paraquat or 2,4-D exposure on testicular physiology were evaluated. In the rats, the exposure doses of paraquat and 2,4-D varied between 0.5 and 30 mg/kg and 75 and 300 mg/kg b.w., respectively, for 2–13 weeks. Exposure to these pesticides (separately) caused histological alterations in the testes, spermatozoid morphological abnormalities, and alteration in reproductive hormones [12,13,14,19,20,52,57,58,59]. However, effects due to short-term exposure to paraquat or 2,4-D on the testes of rats have been scarcely investigated. Paraquat exposure (6, 15, and 30 mg/kg) once daily for five days reduces the sperm count and increases sperm abnormalities. Sperm viability and motility decrease only with high doses [60]. Regarding exposure to 2,4-D, no data were found in short-term exposure (<1 week). This work investigated the effects of short-term exposure (three administrations in one week) to these herbicides on the testes’ thyroid-related genes and ion channels, which are critical molecules involved in reproductive processes.

### 4.1. Effect of Short-Term Exposure to PQT or 2,4-D on the Body, Testes, and Epididymis Weights

Short-term exposure to both herbicides is insufficient for affecting organ weight, as seen in studies with chronic exposure [13,14,19,20,61]. This is likely due to the short exposure time, since at low doses, but in the long term, it can affect the weights of the testicles and epididymis in rats [13,62]. Decreased body weights from exposure to paraquat may be due to reduced food and water intake, malabsorption of nutrients from the gastrointestinal tract, and impaired efficiency in food conversion [20,63].

### 4.2. Effect of Short-Term Exposure to PQT or 2,4-D on Thyroid Signaling in the Testes of Rats

The activity of thyroid hormones is vital for the processes of spermatogenesis and steroidogenesis, directly influencing testicular physiology and therefore male fertility [22,23,24,25]. Cellular factors such as thyroid hormone receptors, deiodinases, and thyroid hormone transporters are required to determine the appropriate action of thyroid hormones for each testicle cell [23,26]. This research showed altered expression of genes associated with the metabolism and transport of thyroid hormones in animals with short-term exposure to both paraquat and 2,4-D pesticides.

Thyroid hormones enter target cells through transporters with varying affinities for each hormone. These transporters are primarily part of the monocarboxylate (MCT) and organic anion transporting polypeptide (OATP) families of cell membrane transporters. Key members with high specificity for thyroid hormones include Mct8, Mct10, and Oatp1c1 [64]. These transporters are expressed in several tissues, including testicular cells [23,65]. Aside from aiding cellular uptake, the Mct8 transporter also helps in the efflux of iodothyronines, making its expression crucial for their metabolism, particularly for T3 (the most active and receptor-affine one). Mct10 is another vital transporter for thyroid hormones—mainly T3—and is less effective for thyroxine T4 but equally as competent as Mct8 [64].

Although PQT exposure did not affect *Mct10* expression, it did increase the expression of *Mct8* and *Oatp1c1* in rat testes. This is contrary to the results for 2,4-D, which only altered the expression of *Mct10* by decreasing it. To date, we have not found any reports on the impact of the herbicides paraquat and 2,4-D on thyroid hormone transporters. However, it is known that acute exposure to paraquat and 2,4-D herbicides can alter thyroid hormone levels [28,66]. Other herbicides, such as glyphosate, can regulate *Mct8* and *Oatp1c1* expression differentially, depending on the organ [41,67].

Deiodinases are enzymes which act by either activating (Dio2) or deactivating (Dio3) the conversion of T4 to T3 to maintain an adequate intracellular concentration of T3 in the target cell. Although *Dio2* expression is detected in the testes of rats, the expression level is known to be relatively low in adulthood. While this research could indicate a decrease in the conversion of T4 to T3 and T3 bioavailability due to treatment with 2,4-D, previous research showed that mice deficient in this enzyme do not exhibit a testicular phenotype, suggesting that they do not play a critical role in testicular development or function [23,64]. On the other hand, the *Dio3* gene showed no changes in gene expression in either herbicide-exposed groups. It is well known that *Dio3* is expressed at high levels during the neonatal testicular stage, decreasing in adulthood [23,68].

Thyroid hormone receptors are transcription factors capable of binding to DNA. Although T4 is the most abundant circulating hormone, T3 is the active hormone, binding with greater affinity to TRs [69]. Gene expression of the thyroid hormone receptor TRα increased in animals exposed to PQT treatment compared with the control group. Regarding treatment with 2,4-D, expression was not affected. TRα is expressed in testicular tissue mostly at early neonatal ages and significantly decreases in adulthood [23]. The functional role of this receptor focuses on mediating thyroid hormone signaling in the testes, Sertoli cells, and Leydig cells, making it vital for their development and steroidogenesis. Therefore, it is necessary to consider that an endocrine disruption mechanism is occurring. Although not in the same way, studies support that TRα expression is increased in a hypothyroid profile [23,41,64]. Interestingly, the gene expression pattern of *Mct8* aligned with the expression pattern of *TRα*, which is highly expressed in Sertoli cells and has been previously reported [70].

The transcription of genes related to thyroid hormones may be due to the autoregulation of hormone levels after herbicide exposure [71]. Differential regulation of thyroid-related genes depends on the type of pesticide, the exposure time, and organ analysis, as has been seen with pentachloroanisole, pentachlorophenol [72], o,p’-DDT, p,p’-DDE [73], glyphosate [41,67], and butachlor [71]. Evaluating the expression of genes related to thyroid hormones can yield biomarkers of early effects since, although the serum levels of thyroid hormones do not change with exposure to herbicides, these genes can be altered [41].

### 4.3. Effect of Acute Administration of PQT or 2,4-D on the Gene Expression of Ion Channels Involved in Flagellar Hyperactivation

This study shows that rats exposed to 100 mg/kg of 2,4-D via an intraperitoneal injection decreased the gene expression of *CatSper1*. These channels are important because they play a role in processes related to sperm capacitation, flagellar hyperactivation, and the acrosomal reaction, which are vital phenomena for fertilization [74]. No significant differences were detected in the expression of *CatSper2* or *Trpv4* in either treatment. The altered expression of *CatSper1* could be associated with the disruption of cellular membrane transport mechanisms and with a decrease in the number of functional channels expressed on the plasma membrane of the sperm, affecting calcium influx, the cell’s ability to maintain ionic gradients, DNA and protein synthesis, as well as the polymerization of microtubules and microfilaments, which could alter the cell shape [75,76]. Although functional studies on sperm are needed to corroborate the results observed in this work, part of the side effects reported due to exposure to 2,4-D (e.g., a decrease in sperm number and motility, as well as an increase in the number of abnormal sperm) [20,21] can be explained by a decrease in the expression levels of *CatSper1*.

Several pesticides, including chlorpyrifos, endosulfan, lindane, cypermethrin, and p,p’-DDE, can interfere with CatSper-mediated Ca^2+^ signaling [37,77]. These pesticides can act as partial agonists or inhibitors of CatSper or exhibit a synergistic effect which alters sperm motility and leads to infertility.

### 4.4. Study Limitations

Despite these significant findings, it is critical to acknowledge the limitations of this study. One of the main limitations is evaluating a single dose of herbicides administered over a short exposure period. Based on the existing literature on the effects of paraquat and 2,4-D on testicular physiology, the herbicide doses selected were 10 mg/kg body weight for paraquat and 100 mg/kg body weight for 2,4-D. However, the single-dose design may not fully capture the range of toxicological effects of these herbicides, especially under different exposure conditions. Exposure to herbicides in real agricultural settings often involves chronic exposure at low doses or repeated acute exposures, which could lead to cumulative impacts on reproductive physiology which were not captured in this study.

Furthermore, environmental factors such as co-exposure to other pesticides or contaminants could exacerbate the reproductive toxicity of paraquat and 2,4-D, complicating the interpretation of studies focusing on individual compounds. Another limitation is that this study focused exclusively on the assessment of gene expression. While gene expression data provide valuable insight into potential mechanisms of herbicide-induced toxicity, further studies are needed to determine how these changes translate into functional alterations in sperm motility, fertility, and overall reproductive success.

## 5. Conclusions

This study provides a first approach to the short-term effects of paraquat and 2,4-D on male reproductive health, focusing specifically on the gene expression of thyroid hormone transporters, deiodinases, receptors, and Ca²⁺-permeable ion channels in the testes of male rats. The findings highlight the potential of these widely used herbicides to disrupt key physiological processes which are critical for fertility and overall reproductive function, even after a brief exposure period.

One of this study’s most significant findings was the differential expression of genes related to thyroid hormone signaling. Paraquat exposure resulted in a marked increase in the expression of the thyroid hormone transporters Mct8 and Oatp1c1, along with a significant upregulation of the TRα receptor in testicular tissue. This suggests that paraquat may exert its toxic effects through endocrine disruption mechanisms, particularly in the metabolism and transport of thyroid hormones, vital spermatogenesis, and steroidogenesis regulators. The increase in TRα expression, a receptor predominantly mediating the effects of thyroid hormones in Sertoli and Leydig cells, suggests that paraquat exposure may induce a compensatory response to maintain thyroid hormone homeostasis in the testes. In contrast, the 2,4-D-treated group showed a decrease in the expression of the Mct10 transporter and the Dio2 deiodinase, indicating possible alterations in the uptake and conversion of thyroid hormones, which could affect T3 bioavailability in testicular cells. Although no previous studies specifically focused on the impact of herbicides like paraquat or 2,4-D on thyroid hormone transporter expression, the current findings build on prior research showing that exposure to these compounds can alter serum thyroid hormone levels. The differential regulation of these transporters and deiodinases highlights a key area for future research, as thyroid hormone signaling is crucial for normal testicular function and male fertility.

Regarding the expression of Ca²⁺-permeable ion channels, specifically those related to sperm motility and hyperactivation, this study revealed significant findings for 2,4-D exposure. A considerable decrease in gene expression of *CatSper1* was observed in the rats exposed to 2,4-D. Since CatSper channels are essential for processes related to sperm capacitation, hyperactivation, and the acrosome reaction, this reduction could have profound implications for sperm function, potentially contributing to decreased motility and abnormal sperm morphology, as reported in previous studies.

### Implications for Future Research

The results of this study underscore the need for further research into herbicides’ molecular and functional effects on male reproductive health. Specifically, future studies should explore the impact of different doses, exposure durations, and combined exposure to other environmental toxins. Additionally, functional assays are essential to validate gene expression findings and determine their impact on fertility. Nevertheless, these assays have limitations as they ignore a pesticide’s systemic effects. One proposal to address this problem is using omics tools, which include the analysis of genomic, proteomic, and metabolomic biomarkers data to elucidate the adverse effects and possible mechanisms of toxicity. Omic biomarkers are promising tools for detecting subclinical effects associated with exposure to environmental pollutants and therefore play an essential role in health risk assessment [78].

Given the widespread use of paraquat and 2,4-D in many countries, understanding their long-term and multigenerational effects on reproductive health is crucial for developing safer agricultural practices and informing public health policies. Although this study is the first approach to the effect of exposure to the herbicides paraquat and 2,4-D through the deregulation of genes related to thyroid hormones and Ca^2+^-permeable ion channels, in proposing that these genes can be considered biomarkers of effect, complementary studies are needed to investigate the effects at the protein and function levels. Furthermore, this assessment should be integrated with other exposure and susceptibility biomarkers to evaluate health risks and properly characterize pesticide impacts.

## Figures and Tables

**Table 1:** Chemical properties of herbicides paraquat and 2,4-D.

Herbicide Name	Paraquat	2,4-D
Chemical Name	1,1′-dimethyl-4-4′-bipyridinium dichloride	2,4-Dichlorophenoxyacetic acid
Molecular Formula	C_12_H_14_Cl_2_N_2_	C_8_H_6_Cl_2_O_3_
Chemical Structure	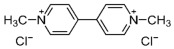	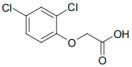
Molecular Weight	257.16 g/mol	221.03 g/mol
LD50 Oral (Rat)	150 mg/kg	639 mg/kg
PubChem CID *	15,938	1486

* https://pubchem.ncbi.nlm.nih.gov/ (accessed on 3 September 2024).

**Table 2:** Primers for PCRs used in this study (forward = F; reverse = R).

Gene Type	Gene	Primer Sequences (5′-3′)	Length (bp)	Reference
Thyroid hormone receptors	*TR* *α*	F: ACCTCCGCATGATCGGGGC R: CCTGATCCTCAAAGACCTC	108	[40,41]
	*TR*β	F: TGGGCGAGCTCTATATTCCA R: ACAGGTGATGCAGCGATAGT	185	
Deiodinases	*Dio2*	F: AGAAGCACCGGAACCAAGAG R: AGCCACAACTTGACACTGGG	89	
	*Dio3*	F: GCCTCTACGTCATCCAGAGC R: GCCCACCAATTCAGTCACTT	170	
Thyroid hormone transporters	*Mct8*	F: CCCAAGCAAGAGAGGCGCCC R: CGGTAGGTGCGCTGGCGAAA	95	
	*Mct10*	F: GGATACTTTGTGCCTTATGTT R: GCAAATAGTCTGCAATGCGG	145	
	*Oatp1c1*	F: GGATCCCCAGTGGGTCGGGG R: ACCAGAAAGGCACGGCTGCA	83	
Ion channels	*CatSper1*	F: TCTTGGAGCGATGAGGAC R: GACGATTGTGTTCAGGCA	204	[42]
	*CatSper2*	F: TGGTTGTTGCTTGGTTCC R: TTCCTTGACTGGTTCCTCT	193	
	*Trpv4*	F: CAGCAAGATCGAGAACCGCCAT R: CGAACTTACGCCACTTGTCCCT	80	[43]
	*Ppia*	F: CCGCTGTCTCTTTTCGCC	129	[40]
		R: GCTGTCTTTGGAACTTTGTCTG		

**Table 3:** Effect of exposure to paraquat or 2,4-D on body, testes, and epididymis weights. Mean ± SD. *** *p* < 0.001.

Organ	CNT (n = 8)	PQT (n = 8)	2,4-D (n = 8)
Weight			
Body	264.5 ± 29.9	197.3 ± 21.5 ***	239.1 ± 36.0
Testes	3.14 ± 0.21	3.10 ± 0.89	3.07 ± 0.46
Epididymis	0.35 ± 0.09	0.28 ± 0.08	0.30 ± 0.09
Somatic Index			
Testes	1.19 ± 0.17	1.59 ± 0.49	1.29 ± 0.12
Epididymis	0.13 ± 0.03	0.14 ± 0.04	0.12 ± 0.03

## Data Availability

The data presented in this study are available in this article.

## References

[1] Hassaan M.A., El Nemr A. (2020). Pesticides Pollution: Classifications, Human Health Impact, Extraction and Treatment Techniques. Egypt. J. Aquat. Res..

[2] Sharma A., Kumar V., Shahzad B., Tanveer M., Sidhu G.P.S., Handa N., Kohli S.K., Yadav P., Bali A.S., Parihar R.D. (2019). Worldwide Pesticide Usage and Its Impacts on Ecosystem. SN Appl. Sci..

[3] Krzastek S.C., Farhi J., Gray M., Smith R.P. (2021). Impact of Environmental Toxin Exposure on Male Fertility Potential. Transl. Androl. Urol..

[4] Mehrpour O., Karrari P., Zamani N., Tsatsakis A.M., Abdollahi M. (2014). Occupational Exposure to Pesticides and Consequences on Male Semen and Fertility: A Review. Toxicol. Lett..

[5] Moshammer H., Poteser M., Hutter H.P. (2020). More Pesticides—Less Children?. Wien. Klin. Wochenschr..

[6] Sukumar C.A., Shanbhag V., Shastry A.B. (2019). Paraquat: The Poison Potion. Indian. J. Crit. Care Med..

[7] Shao X., Li M., Luo C., Wang Y.Y., Lu Y.Y., Feng S., Li H., Lang X.B., Wang Y.C., Lin C. (2015). Effects of Rapamycin against Paraquat-Induced Pulmonary Fibrosis in Mice. J. Zhejiang Univ. Sci. B.

[8] Ardiwinata A.N., Harsanti E.S., Kurnia A., Sulaeman E., Fauriah R., Paputri D.M.W. (2019). Contamination of Paraquat Residues in Soil and Water from Several Provinces in Indonesia. AIP Conf. Proc..

[9] Dorsey E.R., Ray A. (2023). Paraquat, Parkinson’s Disease, and Agnotology. Mov. Disord..

[10] Stuart A.M., Merfield C.N., Horgan F.G., Willis S., Watts M.A., Ramírez-Muñoz F., U J.S., Utyasheva L., Eddleston M., Davis M.L. (2023). Agriculture without Paraquat Is Feasible without Loss of Productivity—Lessons Learned from Phasing out a Highly Hazardous Herbicide. Environ. Sci. Pollut. Res. Int..

[11] Dinis-Oliveira R.J., Duarte J.A., Sánchez-Navarro A., Remião F., Bastos M.L., Carvalho F. (2008). Paraquat Poisonings: Mechanisms of Lung Toxicity, Clinical Features, and Treatment. Crit. Rev. Toxicol..

[12] Li H., Zhu Q., Wang S., Huang T., Li X., Ni C., Fang Y., Li L., Lian Q., Ge R.S. (2019). Paraquat Exposure Delays Stem/Progenitor Leydig Cell Regeneration in the Adult Rat Testis. Chemosphere.

[13] Chen Q., Zhang X., Zhao J.Y., Lu X.N., Zheng P.S., Xue X. (2017). Oxidative Damage of the Male Reproductive System Induced by Paraquat. J. Biochem. Mol. Toxicol..

[14] Li H., Hong T., Zhu Q., Wang S., Huang T., Li X., Lian Q., Ge R.S. (2019). Paraquat Exposure Delays Late-Stage Leydig Cell Differentiation in Rats during Puberty. Environ. Pollut..

[15] Burns C.J., Swaen G.M.H. (2012). Review of 2,4-Dichlorophenoxyacetic Acid (2,4-D) Biomonitoring and Epidemiology. Crit. Rev. Toxicol..

[16] Song Y. (2014). Insight into the Mode of Action of 2,4-Dichlorophenoxyacetic Acid (2,4-D) as an Herbicide. J. Integr. Plant Biol..

[17] Peterson M.A., McMaster S.A., Riechers D.E., Skelton J., Stahlman P.W. (2016). 2,4-D Past, Present, and Future: A Review. Weed Technol..

[18] Panuwet P., Ladva C., Barr D.B., Prapamontol T., Meeker J.D., D’Souza P.E., Maldonado H., Ryan P.B., Robson M.G. (2018). Investigation of Associations between Exposures to Pesticides and Testosterone Levels in Thai Farmers. Arch. Environ. Occup. Health.

[19] Zhou J., Wang H., Jia L., Ma Y., Wang X., Zhu L., Wang K., Zhang P., Yang H. (2022). Mechanism of 2,4-Dichlorophenoxyacetic Acid-Induced Damage to Rat Testis via Fas/FasL Pathway and the Protective Effect of Lycium Barbarum Polysaccharides. Environ. Toxicol..

[20] Marouani N., Tebourbi O., Cherif D., Hallegue D., Yacoubi M.T., Sakly M., Benkhalifa M., Ben Rhouma K. (2017). Effects of Oral Administration of 2,4-Dichlorophenoxyacetic Acid (2,4-D) on Reproductive Parameters in Male Wistar Rats. Environ. Sci. Pollut. Res. Int..

[21] Tan Z., Zhou J., Chen H., Zou Q., Weng S., Luo T., Tang Y. (2016). Toxic Effects of 2,4-Dichlorophenoxyacetic Acid on Human Sperm Function in Vitro. J. Toxicol. Sci..

[22] Krassas G.E., Poppe K., Glinoer D. (2010). Thyroid Function and Human Reproductive Health. Endocr. Rev..

[23] Hernandez A. (2018). Thyroid Hormone Role and Economy in the Developing Testis. Vitam. Horm..

[24] La Vignera S., Vita R., Condorelli R.A., Mongioì L.M., Presti S., Benvenga S., Calogero A.E. (2017). Impact of Thyroid Disease on Testicular Function. Endocrine.

[25] Mazzilli R., Medenica S., Di Tommaso A.M., Fabozzi G., Zamponi V., Cimadomo D., Rienzi L., Ubaldi F.M., Watanabe M., Faggiano A. (2023). The Role of Thyroid Function in Female and Male Infertility: A Narrative Review. J. Endocrinol. Investig..

[26] Romano R.M., Gomes S.N., Cardoso N.C.S., Schiessl L., Romano M.A., Oliveira C.A. (2017). New Insights for Male Infertility Revealed by Alterations in Spermatic Function and Differential Testicular Expression of Thyroid-Related Genes. Endocrine.

[27] Kobal S., Cebulj-Kadunc N., Cestnik V. (2000). Serum T3 and T4 Concentrations in the Adult Rats Treated with Herbicide 2,4-Dichlorophenoxyacetic Acid. Pflugers Arch..

[28] Kongtip P., Nankongnab N., Kallayanatham N., Pundee R., Choochouy N., Yimsabai J., Woskie S. (2019). Thyroid Hormones in Conventional and Organic Farmers in Thailand. Int. J. Environ. Res. Public Health.

[29] Santos R., Piccoli C., Cremonese C., Freire C. (2019). Thyroid and Reproductive Hormones in Relation to Pesticide Use in an Agricultural Population in Southern Brazil. Environ. Res..

[30] Lishko P.V., Kirichok Y., Ren D., Navarro B., Chung J.J., Clapham D.E. (2012). The Control of Male Fertility by Spermatozoan Ion Channels. Annu. Rev. Physiol..

[31] Cong S., Zhang J., Pan F., Pan L., Zhang A., Ma J. (2023). Research Progress on Ion Channels and Their Molecular Regulatory Mechanisms in the Human Sperm Flagellum. FASEB J..

[32] Nowicka-Bauer K., Szymczak-Cendlak M. (2021). Structure and Function of Ion Channels Regulating Sperm Motility—An Overview. Int. J. Mol. Sci..

[33] Wang H.F., Chang M., Peng T.T., Yang Y., Li N., Luo T., Cheng Y.M., Zhou M.Z., Zeng X.H., Zheng L.P. (2017). Exposure to Cadmium Impairs Sperm Functions by Reducing CatSper in Mice. Cell. Physiol. Biochem..

[34] Mohammadi S., Gholamin M., Mohammadi M., Mansouri A., Mahmoodian R., Attari S., Kebriaei S.M., Zibaei B., Roshanaei M., Daneshvar F. (2018). Down-Regulation of CatSper 1 and CatSper 2 Genes by Lead and Mercury. Environ. Toxicol. Pharmacol..

[35] Wang H.F., Liu M., Li N., Luo T., Zheng L.P., Zeng X.H. (2016). Bisphenol a Impairs Mature Sperm Functions by a CatSper-Relevant Mechanism. Toxicol. Sci..

[36] Yuan W.B., Chen H.Q., Li J.Z., Zhou S.M., Zeng Y., Fan J., Zhang Z., Liu J.Y., Cao J., Liu W.B. (2022). TET1 Mediated Male Reproductive Toxicity Induced by Bisphenol A through Catsper-Ca^2+^ Signaling Pathway. Environ. Pollut..

[37] Tavares R.S., Mansell S., Barratt C.L.R., Wilson S.M., Publicover S.J., Ramalho-Santos J. (2013). P,p’-DDE Activates CatSper and Compromises Human Sperm Function at Environmentally Relevant Concentrations. Hum. Reprod..

[38] Zhang X., Kang H., Peng L., Song D., Jiang X., Li Y., Chen H., Zeng X. (2020). Pentachlorophenol Inhibits CatSper Function to Compromise Progesterone’s Action on Human Sperm. Chemosphere.

[39] Mohammadi S., Rahmani F., Hasanian S.M., Beheshti F., Akbari Oryani M., Ebrahimzadeh A., Farzadfar S. (2019). Effects of Dioxin on Testicular Histopathology, Sperm Parameters, and CatSper2 Gene and Protein Expression in Naval Medical Research Institute Male Mice. Andrologia.

[40] Luis E., Fernández Y., Alvarado M., Juárez-Santacruz L., García-Nieto E., Anaya-Hernández A. (2019). Differential Expression and Immunoreactivity of Thyroid Hormone Transporters MCT8 and OATP1C1 in Rat Ovary. Acta Histochem..

[41] de Souza J.S., Kizys M.M.L., da Conceição R.R., Glebocki G., Romano R.M., Ortiga-Carvalho T.M., Giannocco G., da Silva I.D.C.G., Dias da Silva M.R., Romano M.A. (2017). Perinatal Exposure to Glyphosate-Based Herbicide Alters the Thyrotrophic Axis and Causes Thyroid Hormone Homeostasis Imbalance in Male Rats. Toxicology.

[42] Soleimani M.Z., Mashayekhi F.J., Hasanzade M.M., Baazm M. (2018). Alteration in CatSper1 and 2 Genes Expression, Sperm Parameters and Testis Histology in Varicocelized Rats. Int. J. Reprod. Biomed..

[43] Huang X., Hu Y., Zhao L., Gu B., Zhu R., Li Y., Yang Y., Han T., Yu J., Mu L. (2019). TRPV4 Plays an Important Role in Rat Prefrontal Cortex Changes Induced by Acute Hypoxic Exercise. Saudi J. Biol. Sci..

[44] FAOSTAT Pesticides Use. https://www.fao.org/faostat/en/#data/RP/visualize.

[45] Carneiro G.D., de Freitas Souza M., Lins H.A., das Chagas P.S., Silva T.S., da Silva Teófilo T.M., Pavão Q.S., Grangeiro L.C., Silva D.V. (2020). Herbicide Mixtures Affect Adsorption Processes in Soils under Sugarcane Cultivation. Geoderma.

[46] James T.K., Ghanizadeh H., Harrington K.C., Bolan N.S. (2022). The Leaching Behaviour of Herbicides in Cropping Soils Amended with Forestry Biowastes. Environ. Pollut..

[47] Magnoli K., Carranza C.S., Aluffi M.E., Magnoli C.E., Barberis C.L. (2020). Herbicides Based on 2,4-D: Its Behavior in Agricultural Environments and Microbial Biodegradation Aspects. A Review. Environ. Sci. Pollut. Res. Int..

[48] Mostafalou S., Abdollahi M. (2017). Pesticides: An Update of Human Exposure and Toxicity. Arch. Toxicol..

[49] Fritsch C., Appenzeller B., Burkart L., Coeurdassier M., Scheifler R., Raoul F., Driget V., Powolny T., Gagnaison C., Rieffel D. (2022). Pervasive Exposure of Wild Small Mammals to Legacy and Currently Used Pesticide Mixtures in Arable Landscapes. Sci. Rep..

[50] Hamm T.E., King-Herbert A., Vasbinder M.A. (2006). Chapter 27—Toxicology. The Laboratory Rat.

[51] Weber K., Razinger T., Hardisty J.F., Mann P., Martel K.C., Frische E.A., Blumbach K., Hillen S., Song S., Anzai T. (2011). Differences in Rat Models Used in Routine Toxicity Studies. Int. J. Toxicol..

[52] Charles J.M., Cunny H.C., Wilson R.D., Bus J.S. (1996). Comparative Subchronic Studies on 2,4-Dichlorophenoxyacetic Acid, Amine, and Ester in Rats. Fundam. Appl. Toxicol..

[53] Donaher S.E., Van den Hurk P. (2023). Ecotoxicology of the Herbicide Paraquat: Effects on Wildlife and Knowledge Gaps. Ecotoxicology.

[54] Islam F., Wang J., Farooq M.A., Khan M.S.S., Xu L., Zhu J., Zhao M., Muños S., Li Q.X., Zhou W. (2018). Potential Impact of the Herbicide 2,4-Dichlorophenoxyacetic Acid on Human and Ecosystems. Environ. Int..

[55] Chang C., Dai Y., Zhang J., Wu Z., Li S., Zhou Z. (2024). Associations between Exposure to Pesticides Mixture and Semen Quality among the Non-Occupationally Exposed Males: Four Statistical Models. Environ. Res..

[56] Cremonese C., Piccoli C., Pasqualotto F., Clapauch R., Koifman R.J., Koifman S., Freire C. (2017). Occupational Exposure to Pesticides, Reproductive Hormone Levels and Sperm Quality in Young Brazilian Men. Reprod. Toxicol..

[57] Mustafa S., Anwar H., Hussain A., Shabnoor A., Muhammad I., Ijaz U. (2023). Therapeutic Effect of Gossypetin against Paraquat—Induced Testicular Damage in Male Rats: A Histological and Biochemical Study. Environ. Sci. Pollut. Res. Int..

[58] Ijaz M.U., Qamer M., Hamza A., Ahmed H., Afsar T., Abulmeaty M., Ayub A., Razak S. (2023). Sciadopitysin Mitigates Spermatological and Testicular Damage Instigated by Paraquat Administration in Male Albino Rats. Sci. Rep..

[59] Ijaz M.U., Alvi K., Hamza A., Anwar H., Al-Ghanim K.A., Riaz M.N. (2024). Curative Effects of Tectochrysin on Paraquat-Instigated Testicular Toxicity in Rats: A Biochemical and Histopathological Based Study. Heliyon.

[60] D’Souza U.J.A., Narayana K., Zain A., Raju S., Nizam H.M., Noriah O. (2006). Dermal Exposure to the Herbicide-Paraquat Results in Genotoxic and Cytotoxic Damage to Germ Cells in the Male Rat. Folia Morphol..

[61] Oakes D.J., Webster W.S., Brown-Woodman P.D.C., Ritchie H.E. (2002). Testicular Changes Induced by Chronic Exposure to the Herbicide Formulation, Tordon 75D^®^ (2,4-Dichlorophenoxyacetic Acid and Picloram) in Rats. Reprod. Toxicol..

[62] Marty M.S., Neal B.H., Zablotny C.L., Yano B.L., Andrus A.K., Woolhiser M.R., Boverhof D.R., Saghir S.A., Perala A.W., Passage J.K. (2013). An F1-Extended One-Generation Reproductive Toxicity Study in Crl:CD(SD) Rats With 2,4-Dichlorophenoxyacetic Acid. Toxicol. Sci..

[63] Troudi A., Soudani N., Mahjoubi Samet A., Ben Amara I., Zeghal N. (2011). 2,4-Dichlorophenoxyacetic Acid Effects on Nephrotoxicity in Rats during Late Pregnancy and Early Postnatal Periods. Ecotoxicol. Environ. Saf..

[64] Groeneweg S., Van Geest F.S., Peeters R.P., Heuer H., Visser W.E. (2020). Thyroid Hormone Transporters. Endocr. Rev..

[65] Gao Y., Lee W.M., Cheng C.Y. (2014). Thyroid Hormone Function in the Rat Testis. Front. Endocrinol..

[66] Kongtip P., Nankongnab N., Pundee R., Kallayanatham N., Pengpumkiat S., Chungcharoen J., Phommalachai C., Konthonbut P., Choochouy N., Sowanthip P. (2021). Acute Changes in Thyroid Hormone Levels among Thai Pesticide Sprayers. Toxics.

[67] Oliveira J.M., Zenzeluk J., Bargi-Souza P., Szawka R.E., Romano M.A., Romano R.M. (2023). The Effects of Glyphosate-Based Herbicide on the Hypothalamic-Pituitary Thyroid Axis Are Tissue-Specific and Dependent on Age Exposure. Environ. Pollut..

[68] Hernández A. (2018). Thyroid Hormone Deiodination and Action in the Gonads. Curr. Opin. Endocr. Metab. Res..

[69] Dittrich R., Beckmann M.W., Oppelt P.G., Hoffmann I., Lotz L., Kuwert T., Mueller A. (2011). Thyroid Hormone Receptors and Reproduction. J. Reprod. Immunol..

[70] Bae H.S., Jin Y.K., Ham S., Kim H.K., Shin H., Cho G.B., Lee K.J., Lee H., Kim K.M., Koo O.J. (2020). CRISRP/Cas9-Mediated Knockout of Mct8 Reveals a Functional Involvement of Mct8 in Testis and Sperm Development in a Rat. Sci. Rep..

[71] Zhu L., Li W., Zha J., Wang M., Yuan L., Wang Z. (2014). Butachlor Causes Disruption of HPG and HPT Axes in Adult Female Rare Minnow (*Gobiocypris rarus*). Chem. Biol. Interact..

[72] Cheng Y., Ekker M., Chan H.M. (2015). Relative Developmental Toxicities of Pentachloroanisole and Pentachlorophenol in a Zebrafish Model (*Danio rerio*). Ecotoxicol. Environ. Saf..

[73] Wu L., Ru H., Ni Z., Zhang X., Xie H., Yao F., Zhang H., Li Y., Zhong L. (2019). Comparative Thyroid Disruption by o,p’-DDT and p,p’-DDE in Zebrafish Embryos/Larvae. Aquat. Toxicol..

[74] He Y., Wang B., Huang J., Zhang D., Yuan Y. (2024). Environmental Pollutants and Male Infertility: Effects on CatSper. Ecotoxicol. Environ. Saf..

[75] Darszon A., Nishigaki T., Beltran C., Treviño C.L. (2011). Calcium Channels in the Development, Maturation, and Function of Spermatozoa. Physiol. Rev..

[76] Darszon A., Acevedo J.J., Galindo B.E., Hernández-González E.O., Nishigaki T., Treviño C.L., Wood C., Beltrán C. (2006). Sperm Channel Diversity and Functional Multiplicity. Reproduction.

[77] Birch M.R., Johansen M., Skakkebæk N.E., Andersson A.M., Rehfeld A. (2022). In Vitro Investigation of Endocrine Disrupting Effects of Pesticides on Ca^2+^-Signaling in Human Sperm Cells through Actions on the Sperm-Specific and Steroid-Activated CatSper Ca^2+^-Channel. Environ. Int..

[78] Acosta-Tlapalamatl M., Romo-Gómez C., Anaya-Hernández A., Juárez-Santacruz L., Gaytán-Oyarzún J.C., Acevedo-Sandoval O.A., García-Nieto E. (2022). Metabolomics: A New Approach in the Evaluation of Effects in Human Beings and Wildlife Associated with Environmental Exposition to POPs. Toxics.

